# Influence of Chemical Extraction on Rheological Behavior, Viscoelastic Properties and Functional Characteristics of Natural Heteropolysaccharide/Protein Polymer from *Durio zibethinus* Seed

**DOI:** 10.3390/ijms131114871

**Published:** 2012-11-13

**Authors:** Bahareh Tabatabaee Amid, Hamed Mirhosseini

**Affiliations:** Department of Food Technology, Faculty of Food Science and Technology, University Putra Malaysia, 43400 UPM Serdang, Selangor, Malaysia; E-Mail: bahareh.ta@gmail.com

**Keywords:** heteropolysaccharide/protein biopolymer, chemical extraction, decolorizing process, soaking process, protein fraction, rheological properties, viscoelastic behavior

## Abstract

In recent years, the demand for a natural plant-based polymer with potential functions from plant sources has increased considerably. The main objective of the current study was to study the effect of chemical extraction conditions on the rheological and functional properties of the heteropolysaccharide/protein biopolymer from durian (*Durio zibethinus*) seed. The efficiency of different extraction conditions was determined by assessing the extraction yield, protein content, solubility, rheological properties and viscoelastic behavior of the natural polymer from durian seed. The present study revealed that the soaking process had a more significant (*p* < 0.05) effect than the decolorizing process on the rheological and functional properties of the natural polymer. The considerable changes in the rheological and functional properties of the natural polymer could be due to the significant (*p* < 0.05) effect of the chemical extraction variables on the protein fraction present in the molecular structure of the natural polymer from durian seed. The natural polymer from durian seed had a more elastic (or gel like) behavior compared to the viscous (liquid like) behavior at low frequency. The present study revealed that the natural heteropolysaccharide/protein polymer from durian seed had a relatively low solubility ranging from 9.1% to 36.0%. This might be due to the presence of impurities, insoluble matter and large particles present in the chemical structure of the natural polymer from durian seed.

## 1. Introduction

In the last decade, the process development of a new polysaccharide gum from natural sources has attracted attention in biochemistry and pharmacology. This could be due to their sustainability, biodegradability, and biosafety [1]. For instance, guar gum is used for controlling the release of drugs in the gastrointestinal tract [2], and for anticancer drugs in the treatment of colorectal cancer [3]. It is also used in transdermal drug delivery systems [4], and as a supplementation agent in osteoarthritis treatment [5]. In recent years, the physicochemical and functional characteristics of various plant gums have been studied by several researchers: Krueo exudate gum from northwestern Mexican mesquite (*Prosopis* spp.) [6], *Caesalpinia pulcherrima* and *Cassia javanica* seed gum [7,8], mucilage from *Linum usitatissimum* L. seeds [9], mesquite (Prosopis *chilensis* Stuntz) seed [10], and Yanang (*Tiliacora triandra*) leaves [11]. The selection of an appropriate gum is based on certain criteria such as solution clarity, solubility at various temperatures, suspension ability, natural or synthetic, ability to stabilize proteins at a low pH, acid stability, and relative cost per pound [12]. Therefore, it is crucial to investigate the effect of extraction conditions on the physicochemical and functional properties of the gum.

Goycoolea and co-researchers [6] studied the immunological and functional characteristics of the gum from Mexican mesquite (*Prosopis* spp.) seed compared to gum Arabic. They reported the presence of a protein fraction in both mesquite gum and gum Arabic. The researchers found that the mesquite gum showed the proper emulsifying properties but slightly lower than for gum Arabic. They reported that mesquite gum might be a suitable replacement for gum Arabic. Singh and co-workers [8] isolated a water soluble gum from seed endosperm of *Cassia javahikai.* They subjected the crude *Cassia javahikai* gum to several modification processes such as acid fragmentation, methylation, selective enzymatic degradation and periodate oxidation. The researchers found that the grafting modification process led to an increase in the viscosity of *C. javahikai* gum. Singh and co-workers [8] reported that the viscosity of the solution containing *C. javahikai* seed gum decreased when the temperature was increased.

Warrand and co-researchers [9] investigated the structural properties of the neutral polysaccharide from *Linum usitatissimum* L. seeds mucilage. They reported that the weak acid hydrolysis of the side chain led to a reduction in the viscosity of *L. usitatissimum* L. mucilage. Estévez and co-researchers [10] extracted the mucilage from the mesquite (Prosopis *chilensis* Stuntz) seed under different conditions. They found that the acid extraction resulted in lower protein content than the alkaline extraction. This might be due to molecular hydrolysis caused by the acid. The researchers reported that the mesquite (Prosopis *chilensis* Stuntz) seed gum had almost similar protein content to guar gum and locust bean gum (3%–6%). Ibañez and Ferrero [13] investigated the effect of different extraction conditions on the rheological properties of the seed gum from *Prosopis flexuosa* D.C. seed. The researchers reported that the alkalin extraction resulted in a less viscous gum solution than the extraction under natural conditions.

The main goal of the current study was to study the effect of the chemical extraction conditions on the extraction yield, viscosity, protein content and solubility of crude durian seed gum. Three extraction variables namely the decolorizing time (60 min–180 min), soaking time (4 h–12 h) and soaking temperature (25 °C–55 °C) were considered as the independent variables. To the best of our knowledge, there is no published article to report the effect of the chemical extraction conditions on the rheological and functional properties of the natural carbohydrate polymer from durian seed.

## 2. Results and Discussion

### 2.1. Extraction Yield

**Figure 1:**
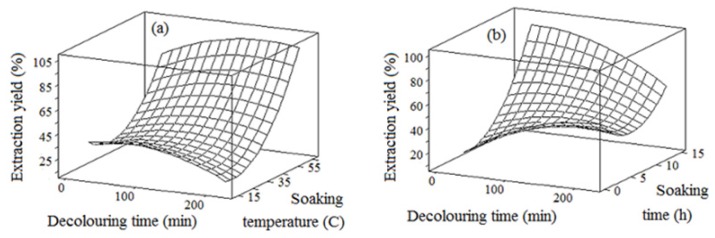
Response surface plots (**a**, **b**) to explain the variation of extraction yield as a function of the chemical extraction conditions.

The results showed that the soaking time followed by the decolorizing time exhibited the most significant (*p* < 0.05) effect on the extraction yield; while the soaking temperature had the least significant (*p* < 0.05) effect on the extraction yield (Table 1). As also shown in Figure 1, the extraction yield differed from 23.7% to 93.8% depending on the chemical extraction conditions. This was higher than the extraction yield reported for hydrocolloid from *Prosopis flexuosa* DC seeds (9%–13%) [13], acid extracted mesquite seed gum (17.7%) and acid extracted mesquite seed gum (24.9%) [10]. Singh and co-researchers [14] reported a relatively low extraction yield (26%) for the mucilage from *Cassis javanica* seed obtained by chemical extraction. It should be noted that the higher extraction yield did not reflect a desirable effect in most cases. For example, the chemical extraction resulting in higher yield led to a crude seed gum with lower solubility. This could be due to the presence of impurities and insoluble matter induced by chemical extraction at an elevated soaking temperature over a long time.

The extraction efficiency also depends on the purity and functional properties of the crude gum extract. Figure 1a,b showed that the extraction yield increased in most cases by prolonging the decolorizing time and soaking time. This could be due to the availability of solvent for a longer time which increased the driving force of mucilage out of the seeds, thereby increasing the extraction yield [15]. The increase in polysaccharide yield could be explained by the large effect of extraction time and temperature on the mass transfer rate of the water soluble polysaccharides in the cell wall [16]. On the other hand, it could be interpreted by the passage of impurities such as pigments or tannic substances from the brown tegument or the seed shell into the crude extract [17]. After soaking in solvent, the endosperm is swollen; therefore the presence of accumulated solvent around the endosperm leads to binding of the soluble components, thus increasing the extraction yield [17].

The chemical extraction at elevated soaking temperature for a longer time led to an increase in the extraction yield (Figure 1a,b). This observation was also reported by previous researchers [18]. This could be due to a reduction in the viscosity of seed mucilage at elevated temperature. Thus, the mucilage can be easily released due to less sticky seeds, thereby increasing the extraction yield. This observation could be also explained by the fact that the solvent molecules have more energy to break the chemical bonds between constituent particles in compounds at elevated temperature, thus increasing the extraction yield [15,16]. The net dissolution process becomes an endothermic reaction (requires energy), if the degree of heating for the dissolution process is less than the heating level required to rupture the solid. Under this condition, elevated temperature facilitates the dissolution process by giving more energy to break apart the bonds in the solid. As explained by Piñeiro and co-researchers [19], elevated temperature results in a higher extraction yield due to two different mechanisms. First, elevated temperature results in weakness of the bonds between the polysaccharide gum and its matrix, thereby increasing its solubility. Second, the high extraction temperature results in the degradation of compounds. Therefore, elevated temperature is a driving force facilitating the extraction of the degraded compounds. Although elevated temperature is favourable for the extraction of carbohydrates, the thermal degradation however, induces undesirable effects on the functional properties.

### 2.2. Viscosity

The results indicated that the viscosity of crude durian seed gum varied from 3.3 mPa.s to 24.3 mPa.s depending on the extraction conditions. The changes of viscosity could be related to the significant (*p* < 0.05) effect of the soaking process on the content and structure of the protein fraction present in the structure of the gum. The viscosity of a hydrocolloid is dominated by concentration, molecular weight and structure (linear, branched or slightly branched). In general, durian seed gum did not induce a very high viscosity after dispersing in water. This might be due to the fact that durian seed gum does not have a high molecular weight (*M*w) structure as reported previously [20]. It has been proven that the gum with a larger *M*w causes stronger viscosity than the gum with a lower Mw. Our previous study [20] revealed the considerable effect of extraction conditions on the molecular structure of durian seed gum. This might be responsible for significant changes in the apparent viscosity of the gum as a function of the extraction variables. In addition, the apparent viscosity also depends on the chemical composition and structure of the gum. As also reported earlier [20], the extraction conditions significantly affected the chemical structure (*i.e.*, carbohydrate, protein and lipid fraction) of the crude durian seed gum. This could be also responsible for significant changes in the apparent viscosity of durian seed gum as a function of extraction variables. As also stated by Balaghi and co-researchers [21], several factors (such as neutral sugars, uronic acid, methoxyl group content, configuration and position of glycosidic linkages, molecular weight and conformational properties) play a crucial role in the rheological properties of the hydrocolloid.

In the current study, the range of viscosity was lower than the viscosity reported for *Prosopis flexuosa* DC seed mucilage (58 mPa.s–277 mPa.s at a shear rate of 64 1/s) [13], achi (*Brachystegea eurycoma*), and basil seed gum (581.37 mPa.s at a shear rate of 98.9 1/s) [22]. However, the rheological properties of hydrocolloid depend on shear rate, temperature, pressure and shearing time [23]. As stated by previous researchers [24], the rheological properties of a hydrocolloid are mainly influenced by the droplet size and distribution, droplet volume fraction, droplet concentration, deformability and the inter-droplet forces. The chemically-extracted crude durian seed gum showed a typical “pseudoplastic or shear-thinning behavior”. The viscosity of the sample decreased with increasing shear rate. As the shear rate increased, the droplet–droplet interaction became deformed and eventually disrupted, thus resulting in a size reduction of the flocks thereby decreasing the viscosity [25]. Previous researchers also reported a similar shear-thinning behavior in the following plant gums: basil seed gum [3], cress seed (*Lepidium sativum*) gum [26], and *Alyssum homolocarpum* seed gum [27].

**Figure 2:**
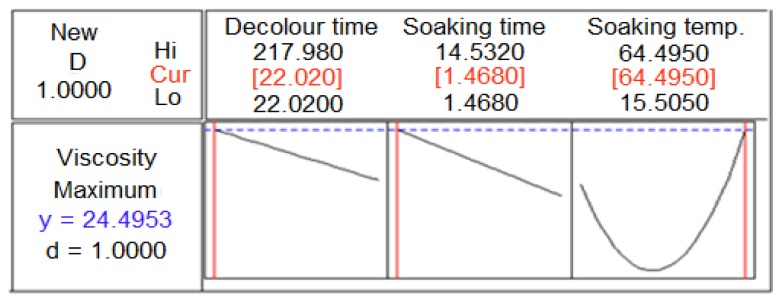
Single response optimizer plot for explaining the variation of viscosity as a function of the chemical extraction conditions.

The soaking temperature and decolorizing time exhibited the most and least significant (*p* < 0.05) effect on the viscosity of the crude durian seed gum. The present study revealed that the viscosity increased as the extraction was carried out for a short decolorizing and soaking time (Figure 2). In general, the viscosity of crude durian seed gum solution increased when the soaking temperature was increased (Figure 2). This might be explained by the more efficient extraction of polysaccharides at the elevated soaking temperature which increased the viscosity. On the other hand, the extraction at an elevated soaking temperature led to an increase in the total soluble solids, thus inducing a higher thickness. This is in agreement with that reported by Ozkanli [28]. Singthong and his co-researchers [11] also reported that the extraction at elevated temperature and extended time provided a high extraction yield, which was responsible for a high viscosity for Yanang gum.

### 2.3. Protein Content

**Figure 3:**
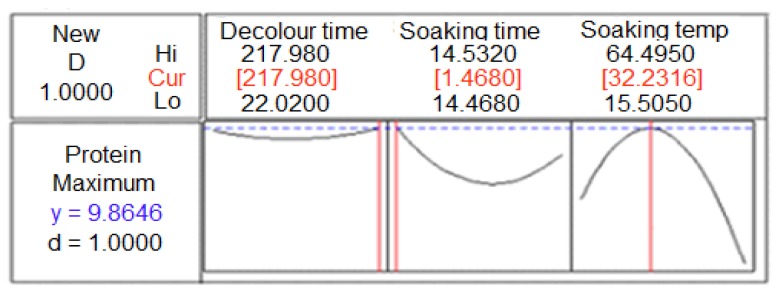
Single response optimizer plot to explain the variation of protein content as a function of chemical extraction conditions.

The protein content is a key biological aspect of a gum. The presence of protein in polysaccharides can induce an inflammatory response in tissues, thereby inhibiting the pharmacological application of polysaccharide-based materials [29]. Our previous study [20] revealed that the protein fraction and amino acid composition of durian seed gum were significantly influenced by extraction variables which had a significant (*p* < 0.05) effect on the protein content of durian seed gum. As shown in our previous study [20], the most abundant amino acids of durian seed gum were leucine (30.9%–37.3%), lysine (6.04%–8.36%), aspartic acid (6.10%–7.19%), glycine (6.07%–7.42%), alanine (5.24%–6.14%), glutamic acid (5.57%–7.09%), valine (4.5%–5.50%), proline (3.87%–4.81%), serine (4.39%–5.18%), threonine (3.44%–6.50%), isoleucine (3.30%–4.07%), and phenylalanine (3.11%–9.04%). The results indicated that the soaking process led to a reduction in the protein content when the extraction was carried out at elevated temperature (>36 °C) and prolonged time (Figure 3).

The “protein fraction” can be responsible for the surface activity and this hydrophobic nature is an obligatory requirement for gums to adsorb onto interfaces. The present study revealed that the chemical extraction under the medium soaking temperature (<36 °C) resulted in low thermal denaturation of the protein fraction present in the gum structure. This observation was also reported by Abd El-Hady and Habiba [30]. They reported that the soaking process decreased the protein level of the crude gum. They concluded that the soaking process resulted in the leaching of some of the water-soluble proteins from the gum structure into the soaking medium. They revealed that the soaking treatment led to an improvement in the *in vitro* digestibility, enhanced the phosphorus availability (by reducing phytate and phytate phosphorus), and reduced tannins, polyphenols, and α-amylase inhibitors.

In the current study, the protein content of crude durian seed gum ranged from 3.8% to 9.3%, depending on the chemical extraction conditions. This value was almost similar to the protein content reported for *Opuntia ficus* indica (7.3%) [25], crude locust bean gum (4.3%–5.0%) [31], and malva nut gum (6.7%) [32]; while it was lower than that reported for *Prosopis flexuosa* seed gum (10%–20%) [13]. On the other hand, it was greater than the protein content reported for basil seed gum (2.17%) [3], Mesquite seed gum (2.5%) [10], cress seed gum (0.6%) [26], and Krueo Ma Noy pectin (3.29%) [33]. According to Glicksman [34], the protein content in commercial guar gum, locust bean gum and Tara gum was found to be 10%, 8% and 3.13%, respectively. The results indicated that the protein content decreased by prolonging the soaking time from 1.46 h to 6.90 h. Conversely, it increased when the chemical extraction was carried out for a longer soaking time (>7 h). However, the short soaking process (1.46) resulted in the highest protein content in the studied range of soaking times (Figure 3). On the other hand, the reverse trend was observed with the soaking temperature. As shown in Figure 3, the protein content increased with increasing temperature from 15.5 °C to 36 °C. As also reported by Koocheki and co-researchers [27], the protein content was increased due to the greater rate of mass transfer at the higher temperature. Conversely, the further increase in soaking temperature led to a reduction in the protein content. The protein reduction could be explained by thermal denaturation caused by the relatively high soaking temperature.

Adewusi and Osuntogun [35] illustrated that the soaking process for a short time (<30 min) would promote nutrient retention by maintaining a higher content of protein; while the soaking process at high temperature (90 °C) for a long time (>30 min) led to continuous reduction of the protein and total solids. Above 32 °C, protein retention was observed. Protein retention reached a maximum at a temperature of 60 °C which was associated with the denaturation of the protein system, heat-induced Millard response and protein–protein interaction [35]. This could also account for a reduction in the protein content of crude durian seed gum when the soaking process was carried out at an elevated temperature (>36 °C). The results indicated that the interaction between the chemical extraction variables did not show any significant (*p* > 0.05) effect on the protein content (Table 2). In the current study, the decolorizing time exhibited an insignificant (*p* > 0.05) effect on the protein content (Table 2). It should be noted that the protein content was more significantly (*p* < 0.05) influenced by the soaking time rather than the soaking temperature (Table 2). In fact, the soaking time had the most significant (*p* < 0.05) effect on the protein content (Table 1). Therefore, it should be considered to be the most critical factor affecting the protein content present in the molecular structure of the gum.

### 2.4. Solubility

**Figure 4:**
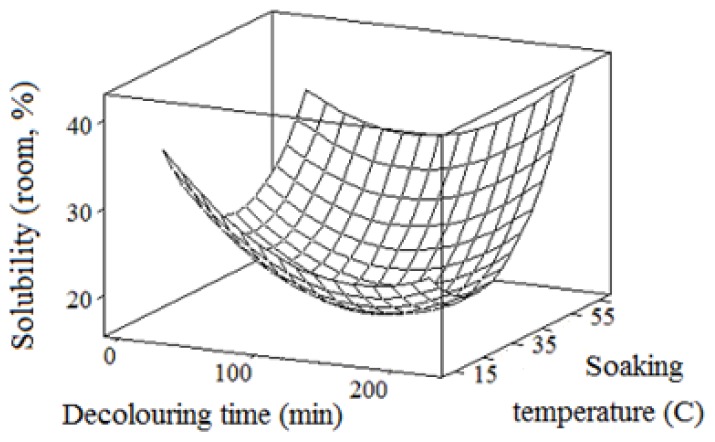
Response surface plot to explain the variation of solubility as a function of the chemical extraction conditions.

The present study revealed that the natural polymer from durian seed had a relatively low solubility ranging from 9.1% to 36.0% (Figure 4). This was less than the solubility reported for carob gum (~50%) [36], xanthan gum and guar gum (>40%) [37]. On the other hand, the crude durian seed gum showed an almost similar solubility to *Lepidium Perfoliatum* seed gum (18%–25%), and locust bean gum (30%) [37]. The results indicated that durian seed gums extracted under different experimental conditions showed different degrees of solubility. This might be due to the significant (*p* < 0.05) effect of the extraction conditions on the chemical and molecular structure of durian seed gum. In fact, the different degree of solubility might be explained by the significant effect of extraction conditions on the carbohydrate composition of durian seed gum. As shown in our previous study [20], galactose was the most abundant monosaccharide in the carbohydrate composition of durian seed gum. It should be noted that galactose content plays a significant role in the solubility of various plant gums. A plant gum containing more galactose has better solubility than a gum with lower galactose content. In addition, the carbohydrate analysis revealed the presence of a high content of glucose and a low percentage of arabinose and xylose in the chemical structure of durian seed gum. Our previous study [20] reported the significant effect of extraction variables on the monosaccharide composition (*i.e.*, galactose, glucose, arabinose and xylose) of durian seed gum, thereby affecting its solubility.

On the other hand, the low water solubility could be due to the presence of impurities (*i.e.*, husk, germ fractions), insoluble matter and large particles present in the crude gum [38]. Our previous study [20] also revealed the presence of protein and lipid fraction in the chemical structure of both crude and purified durian seed gum. In fact, different purification techniques did not exclusively remove the protein and lipid fraction from the structure of durian seed gum. This might be responsible for the low solubility of the natural polymer from durian seed. Westphal and Jann [39] stated that the protein fraction was more soluble in phenol and the polysaccharide was more soluble in water. Therefore, the polysaccharide fraction containing high protein content showed low solubility. It was hypothesized that the considerable effect of extraction conditions on the amino acid composition and fatty acid profile of durian seed gum might be another reason for the different degree of solubility. As reported earlier [20], the extraction conditions significantly influenced the amino acid composition of the protein fraction present in the chemical structure of durian seed gum. The different degree of solubility might be also due to the significant effect of extraction conditions on fatty acid composition of durian seed gum [20]. Our previous study [20] also reported the presence of saturated and unsaturated fatty acids (*i.e.*, palmitic acid (C 16:0), palmitoleic acid (C 16:1), stearic acid (C 18:0), oleic acid (C 18:1), linoleic acid (C 18:2), and linolenic acid (C 18:2)) in the chemical structure of durian seed gum. Our preliminary study indicated that the crude gum containing a lower content of the hydrophobic lipid fraction, saturated fatty acid (*i.e.*, palmitic acid (C 16:0) and stearic acid (C 18:0)) showed a higher solubility than the crude gum.

In addition to chemical composition, the molecular structure of durian seed gum was also influenced by extraction conditions, as reported in our previous study [20]. The crude gum with a larger molecular weight (*M*w) and particle size has more difficulties in the water absorption and solubilization process. Our preliminary study indicated that the crude gum with lower solubility had a larger particle size than the crude gum with a smaller particle size. Pollard and co-workers [38] also reported that the solubility of carob galactomannan did not exceed 50%. They explained that this could be due to the presence of large particles in the crude carob gum. In the current study, both decolorizing and soaking variables significantly (*p* < 0.05) affected the solubility of crude durian seed gum. The solubility was significantly (*p* < 0.05) influenced by the main effects of all extraction variables and the interaction effect of soaking time and temperature (Table 2).

Hydrocolloids behave differently from common food ingredients due to their hydration process [40]. In ordinary food ingredients such as sucrose, when subjected to water, they dissolve starting with the outside layer. Under this condition, the size decreases and they are fully dissolved in water. When hydrocolloids are added to water, they absorb water and swell like a sponge [41]. The water solubility of a homogenous polysaccharide is different from a polysaccharide-protein structure. The natural polymer from durian seed is a heterogeneous polysaccharide plus protein fraction; therefore, its solubility depends on the interaction between the monosaccharide and the protein fractions in the aqueous system. According to Tolstoguzov [42], the protein-polysaccharide structure may lead to three different interaction patterns in the solution: (I) compatible or co-solubility, (II) association or complex coacervation and (III) incompatibility. In association, the phase separation of both biopolymers from the phase containing solvent takes place during the complex coacervation. This occurs because of the electrostatic interaction between differently charged biopolymers [43,44]. However, the most usual pattern corresponds to the thermodynamic incompatibility between protein and polysaccharide molecules, thus reducing the solubility of the protein-polysaccharide mixture in the aqueous solution. Thermodynamic incompatibility occurs because the interaction between similar biopolymers is energetically more favorable than the interaction between different biopolymers [45]. This incompatibility results in the formation of two separated phases [44,46].

The soaking temperature had the most significant (*p* < 0.05) effect on the solubility (Table 2). In fact, the neutral gums are less soluble than those containing uronic acids, which are often in the polyelectrolyte form (charged or ionic). When the hydrocolloids dissolve in water, they give different conformations (spherical, random coil or rod-like). During the soaking process, the seed absorbs moisture, swells up and some water- soluble nutrients leach into the soaked water by simple diffusion. This swelling behavior depends on the nature and ratio of the matrix and solvent, the soaking temperature and the length of soaking time [47]. A relatively wide range of solubility could be also due to the presence of different protein content. In fact, the water solubility of a homogenous polysaccharide is different from a polysaccharide-protein structure (or heterogeneous polysaccharide).

As also stated by Laaman [48], particle size is also a fundamental issue affecting the solubility. The larger particles correspond to a coarser mesh size which take longer to dissolve due to the longer time required for the water to penetrate into the matrix. Whereas a smaller particle takes a shorter time to penetrate the water and become fully soluble [48]. Koocheki and co-researchers [37] also reported the maximum solubility (25%) of *Lepidium perfoliatum* seed gum. They described that the low solubility of *Lepidium perfoliatum* seed gum might be related to the presence of impurities in the chemical structure of the crude gum. A natural gum may not be fully dissolved in water due to the presence of insoluble particles [48]. Therefore, further purification is required to reduce the insoluble matters and consequently enhance the solubility of the crude gum.

### 2.5. Elastic Modulus (G′) and Viscous Modulus (G″)

The elastic modulus (*G′*) and viscous modulus (*G″*) are the critical parametric data in the oscillation test. The elastic modulus (*G′*) is an indicator of the deformation energy accumulated in the sample during the shear process, thus representing the gel or solid behavior of the sample. The viscous modulus (*G″*) is an indicator of the deformation energy accumulated in the sample during the shear and lost to the sample, showing the liquid behavior of the sample [49]. If the elastic modulus (*G′*) is higher than the viscous modulus (*G″*), the sample behaves more like a solid with gel like behavior and weak elastic behavior. As stated by Simas-Tosin and co-workers [50], the elastic modulus (*G′*) is related to the solid response of the material and viscous modulus (*G″*) is related to the fluid response of the material. Therefore, a sample with higher elastic value (*G′*) has more rigidity than the sample with lower *G′*[51]. Conversely, if the elastic modulus (*G′*) is lower than the viscous modulus (*G″*), the energy used to deform the material is dissipated viscously, and the sample behaves like a liquid [49].

**Figure 5:**
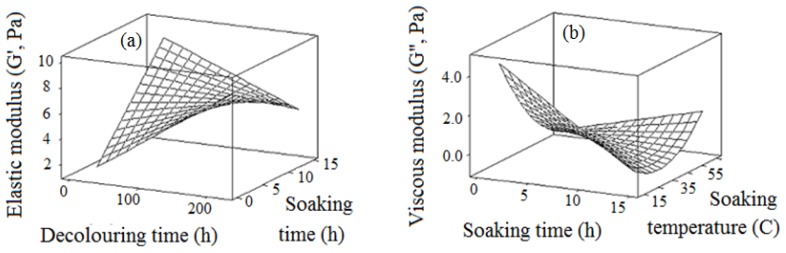
Response surface plot (**a**, **b**) to explain the variation of elastic modulus (*G′*) and viscous modulus (*G″*) as a function of chemical extraction conditions.

In the current study, the degree of elastic modulus (*G′*) varied from 1.48 Pa to 9.46 Pa; while the degree of viscous modulus (*G″*) ranged from 0.28 Pa to 4.51 Pa (Figure 5a,b). This observation indicated that the chemically-extracted durian seed gum showed more elastic behavior rather than viscous behavior at the low frequency (0.1 Hertz). In fact, chemically-extracted durian seed gum had a typical gel network. However, if the ratio of viscous modulus (*G″*) to elastic modulus (*G′*) (tan δ) is greater than 0.1, it means that the sample is not a true gel [51]. This sample has a structure between a concentrated biopolymer and a true gel, thus indicating weak gel like behavior. In this sample, chain entanglements and macromolecule interconnections are not permanent; therefore, they could be disrupted by inducing high shear rates [51]. This confirms the pseudoplasticity of crude durian seed gum in the steady state experiments. This observation was also reported by Mandala and co-researchers [51] for locust bean gum and xanthan gum.

The degree of elastic modulus (*G′*) of crude durian seed gum was similar to that reported by Wang and co-researchers [52] for 0.5% flaxseed gum (3.5 Pa–5.5 Pa); while it was less than 3% maize starch (MS) gel (9.8 Pa–27.9 Pa) [52], xanthan gum 0.15% and locust bean 0.09% (>100 Pa) [51] at low frequency. Figure 5a also showed that the low elastic behavior was achieved by reducing the decolorizing time and prolonging the soaking time. Table 2 demonstrated that soaking time and decolorizing time exhibited the most and least significant (*p* < 0.05) effect on the elastic modulus (*G′*). As shown in Figure 5b, the degree of viscous behavior (*G″*) increased when the soaking time and temperature decreased. The results indicated that the soaking process for a short time at low temperature led to an increase in the viscous behavior (*G″*) of crude durian seed gum.

The degree of viscous modulus (*G″*) of crude durian seed gum ranged from 0.28 Pa to 4.51 Pa. It was similar to *G″* reported for 3% maize starch (MS) gel (0.8 Pa–6.2 Pa) and 0.5% flaxseed gum (0.4 Pa–3.1 Pa) [52]. In general, the chemically-extracted durian seed gum showed solid (gel like) behavior at low concentration (0.5%, *w*/*w*) and low frequency (0.1 Hertz). However, the degree of solid (or gel like) or liquid (or viscous) behavior depends on the gum concentration and frequency measurement. Wang and co-workers [52] found that the flaxseed gum showed gel-like behavior at low concentration (0.5%), since the elastic modulus (*G′*) was extremely greater than the viscous modulus (*G″*). They also reported that both the elastic modulus (*G′*) and viscous modulus (*G″*) of flaxseed gum increased with increasing gum concentration. The precipitation in alcohol and soaking in acetic acid yielded a less viscous solution. As shown in Table 2, the soaking temperature exhibited the most significant (*p* < 0.05) effect on the viscous modulus. In general, the viscous behavior of durian seed gum was more significantly influenced by the soaking process rather than the decolorizing process.

## 3. Experimental Section

### 3.1. Materials and Methods

Durian fruits (*D. zibethinus*) were purchased from the local market (Selongor, Malaysia). They were selected based on size uniformity and free of visual defects. Initially, the ripened fruits were de-husked by cutting along the suture on the back of the lobules, and their seeds were removed, cleaned, and rinsed thoroughly with sterile distilled water. The washed fruit seeds were partially dried by air circulation due to prevent the germination and prolong the storage life. They were then packed in plastic bags and stored in a dry and cool place (10 °C ± 2 °C) until the extraction process. In addition, glacial acetic acid (≥99.7%), methanol, hexane, isopropanol, petroleum ether, ethanol (95%) and absolute ethanol (99.9%) were purchased from Fisher Scientific (Leicestershire, UK).

### 3.2. Chemical Extraction

Chemical extraction of durian seed gum was carried out according to the method described by Singh and co-researchers [14] with a minor modification. Initially, durian seeds were chopped into small pieces and air dried before milling into the seed flour. Then, the seed flour was subjected to the defatting process to extract the lipid fraction from the seed flour. The defatting process was carried out successively using hexane and isopropanol (60:40) at room temperature (25 °C ± 1 °C). The solvent residue was eliminated through the centrifugation at 1400*g* for 15 min (Avanti J-25 Centrifuge, Krefeld, Germany). Then, defatted seed flour (1 kg) was exhaustively decolorized using ethanol at different decolouring times (60 min–180 min). Subsequently, the decolourized seed flour was filtered and then soaked in 1% aqueous acetic acid under different soaking conditions (times, 4 h–12 h; temperatures, 25 °C–55 °C). Then, the slurry was filtered with a Muslin cloth filter and the filtrate was precipitated with 95% ethanol [53]. The precipitate was washed successively with acetone/ether (1:1), and then kept in a fume hood at room temperature overnight. The crude gum was freeze dried at −50 °C for 48 h using a freeze drier (Labconco Freezone 18, Model 77550, Kansas City, MO, USA). It should be noted that the homogenized-gum solution (10%) was pre-frozen at −20 °C for 24 h prior to freeze-drying process. The chemical extraction was carried out in duplicate for each extraction run.

### 3.3. Analytical Tests

#### 3.3.1. Extraction Yield

The extraction yield of the natural polymer from durian seed was determined by weighing the dried extract and calculating the wet basis as reported by previous researchers [15]:

(1) $$Y=100\times (M_{1}/M_{2})$$

where *M*_1_ and *M*_2_ are the mass of extracted gum and durian seed, respectively.

#### 3.3.2. Measurement of Protein Content

The protein content of the natural polymer from durian seed was determined by measuring the nitrogen content (micro-Kjeldahl). The protein content was calculated from the nitrogen content by using a conversion factor of 6.25. The experiment was performed in triplicate by using a rapid distillation system (VAPODEST 20, rapid distillation system, Gerhardt GmbH & Co, Brackley, Northants, UK). Approximately 1 g of the crude polymer and 12 mL–15 mL of concentrated sulfuric acid were placed into a flask. Then, 7 g of potassium sulfate and a copper catalyst were added to the mixture, and the mixture was heated up to 40 °C for 60 min. In the next step, 50 mL of hot deionized water was added to the mixture after cooling to 20 °C. Sodium hydroxide solution (45%, *w*/*v*) was mixed to convert ammonium ions to ammonia gas at the higher pH. The ammonia gas was trapped by 1 M boric acid solution, and titrated with 5 mM sulfuric acid to neutralize using methyl red and methylene blue as indicators. The protein analysis was reported based on dry basis according to the following equation [54]:

(2) $$\begin{array}{c} 1\;\text{mL of}\;\text{sulfuric acid }(5\;\text{mM})=0.14007\;\text{mg}\;\text{nitrogen}\\ \text{Protein content }(\%)=\text{Nitrogen content }(\%)\times 6.25 \end{array}$$

#### 3.3.3. Solubility Measurement

The solubility was measured according to the method described by previous researchers [36]. In this experiment, 1 g of the crude polymer was added to 100 mL of distilled water and agitated with a stirrer for 30 min at ambient temperature (25 °C ± 1 °C). Then, it was subjected to centrifugation at 6000*g* for 30 min to remove insoluble material. The supernatant was transferred to disposable Petri dishes and oven dried at 105 °C for 24 h to constant weight. The percent solubility was calculated by the weight difference and expressed as dry basis. The solubility measurement was carried out in triplicate and the average of three individual measurements was considered for further data analysis.

#### 3.3.4. Determination of Apparent Viscosity

The apparent viscosity of the polymer from durian seed was determined by using a Haake rheometer (RheoStress 600, Karlsruhe, Germany). The shear stress/shear rate rotation ramp test (mechanical spectra) was performed using a cone sensor (C35/2° Ti; 222–1632; 35 mm diameter, 2° angle), with 0.105 mm gap and a measuring plate cover (MPC 35; 222–1549). Initially, 0.5 g of the crude polymer was dissolved in water to obtain the gum solution (0.5%, *w*/*w*), then it was agitated robustly for approximately 15 min until dissolved. Finally, it was stirred overnight to form a homogenous viscous dispersion. Two milliliter of the gum solution (0.5%, *w*/*w*) was placed on the rheometer plate and allowed to equilibrate for 10 min at 25 °C. The viscosity measurements were carried out in triplicate after 24 h of hydration of the gum solution at a shear rate of 10–200 s^−1^[55].

#### 3.3.5. Dynamic Rheological Measurements

Dynamic rheological properties (*i.e.*, elastic or storage modulus, *G′*; viscous or loss modulus, *G″*) of the polymer from durian seed were determined by using a Haake rheometer (RheoStress 600, Haake, Karlsruhe, Germany). The dynamic Haake rheometer equipped with a cone and plate probe was applied to the test strain amplitude sweep at a fixed frequency of 0.1 Hz and 25 °C isothermal conditions. The measurement was performed under a small amplitude oscillatory shear mode using parallel plate geometry (25 mm diameter). The amplitude of strain was swept from 0.02 Pa to 1 Pa with the increment of a logarithmic scale [56]. The specimen was allowed to equilibrate at the measurement conditions for approximately 10 min prior to each sweep run. The oscillatory test was measured in triplicate in order to check the reproducibility of the measurements.

### 3.4. Experimental Analysis

Response surface methodology was applied to investigate the effect of three extraction variables namely decolorizing time, soaking time and soaking temperature on physicochemical and functional properties of gum from durian seed. A central composite design (CCD) including six centre points, six axial points and eight factorial cube points was used to create twenty treatments (Table 3). Polynomial multiple regression (PMR) was applied to describe the variation of the functional properties of durian seed gum as a function of three extraction variables. Significant differences were evaluated by PMR at the 95% confidence level. The creation of experimental design and further data analysis was carried out by using Minitab V. 15 (Minitab Inc., Pine Hall Rd, State College, PA, USA).

## 4. Conclusions

The current work revealed that the crude natural polymer from durian seed gum had relatively low solubility, thus minimizing its functional applications. The present study indicated that the extraction process with a high recovery level did not always provide the appropriate functional properties. This could have been due to the presence of a higher impurity content and insoluble matter which resulted in the low solubility of the crude durian seed gum. Therefore, a further purification process is recommended to minimize the impurities and consequently improve the solubility and other functional properties of the crude gum. The results indicated that the extraction yield, viscosity, and solubility increased when the extraction was carried out at an elevated soaking temperature. This could be due to thermal degradation of the protein fraction at the elevated extraction temperature, thereby affecting the solubility and other functional properties of the crude gum. The current study revealed that the chemical extraction at an elevated soaking temperature (>36 °C) led to a reduction in the protein content, providing purer gum with probably a more appropriate biological function than the gum extracted at the low temperature. However, extraction at a very high temperature, which results in the thermal degradation of the gum structure, is not recommended.

## Figures and Tables

**Table 1:** Regression coefficients, *R^2^*, *p*-value and lack of fit for the final models.

Regression coefficient	Extraction Yield (%)	Viscosity (mPa.s)	Protein Content (%)	Elastic modulus *G′* (Pa)	Viscous modulus *G″* (Pa)	Solubility (%)
*b*_0_	45.021	15.800	1.0133	0.000190	0.45255	56.401
*b*_1_	0.318	0.165	–	0.000002	–	−0.235
*b*_2_	−1.623	3.493	−0.8273	0.000034	−0.02514	0.415
*b*_3_	−2.527	−1.704	0.5260	–	−0.01420	−1.487
*b*_1_^2^	−0.001	−0.001	–	–	–	0.001
*b*_2_^2^	0.515	−0.216	0.0455	–	–	–
*b*_3_^2^	0.041	0.024	−0.0074	–	0.00010	0.018
*b*_12_	−0.029	–	–	−0.000000	–	–
*b*_13_	0.004	–	–	–	–	0.002
*b*_23_	–	–	–	–	0.00049	–
*R*^2^	0.988	0.879	0.952	0.763	0.882	0.975
*p* value	0.000 *	0.005 *	0.000 *	0.048 *	0.000 *	0.001 *
Lack of fit (*p* value)	0.122	0.827	0.765	0.676	0.000 *	0.622

*b*_1_, *b*_2_ and *b*_3_: the estimated regression coefficients for the linear effects; *b*_1_^2^, *b*_2_^2^ and *b*_3_^2^: the estimated regression coefficients for the quadratic effects; *b*_12_, *b*_13_ and *b*_23_: the estimated regression coefficients for the interaction effects; 1: decolorizing time; 2: soaking time; 3: soaking temperature;

* : significant (*p* < 0.05).

**Table 2:** *p*-value and *F*-ratio of chemical extraction variables in final reduced models.

Independent variable terms		Extraction Yield (%)		Viscosity (mPa.s)		Protein content (%)		Solubility (%)		Elastic modulus (*G′*, Pas)		Viscous modulus (*G″*, Pas)	
					
		*p*	*F*-ratio	*p*	*F*-ratio	*p*	*F*-ratio	*p*	*F*-ratio	*p*	*F*-ratio	*p*	*F*-ratio
Main effects	*x*_1_	0.024	8.22	0.018	8.84	–	–	0.001	29.76	0.025	6.677	–	–
	*x*_2_	0.301 *	1.24	0.003	17.58	0.000	48.34	0.010	11.41	0.011	9.302	0.000	40.602
	*x*_3_	0.057 *	5.16	0.001	27.95	0.000	35.10	0.000	42.38	–	–	0.000	35.784

Quadratic effects	*x*_11_	0.028	7.64	0.019	8.532	–	–	0.001	22.33	–	–	–	–
	*x*_22_	0.000	40.43	0.003	17.93	0.000	39.05	–	–	–	–	–	–
	*x*_33_	0.019	9.13	0.001	30.70	0.000	45.45	0.000	40.04	–	–	0.002	17.859

Interaction effects	*x*_12_	0.001	31.99	–	–	–	–	–	–	0.029	6.340	–	–
	*x*_13_	0.027	7.70	–	–	–	–	–	–	–	–	–	–
	*x*_23_	–	–	–	–	–	–	0.049	5.39	–	–	0.000	29.246

* Non-significant at *p* > 0.05;

*p: p*-value; *x*_1_, *x*_2_ and *x*_3_ represents the main or single effect of decolorizing time, soaking time and soaking temperature, respectively; *x*_1_^2^, *x*_2_^2^ and *x*_3_^2^ represents the quadratic effect; *x*_1_*x*_2_, *x*_1_*x*_3_ and *x*_2_*x*_3_ represents the interaction effects of extraction variables, respectively.

**Table 3:** Central composite design including independent and response variable.

Variable	Independent variable levels				
Independent Variables	Low	Center	High	Axial (−α)	Axial (+α)
Decolorizing time (*x*_1_, 60 min–180 min)	60	120	180	22	218
Soaking time (*x*_2_, 4 h–12 h)	4	8	12	1.5	14.5
Soaking temperature (*x*_3_, 25 °C–55 °C)	25	40	55	15.5	64.5

## References

[1] Dodi G., Hritcu D., Popa M.I. (2011). Carboxymethylation of guar gum: Synthesis and characterization. Cellul. Chem. Technol.

[2] Chourasia M.K., Jain S.K. (2004). Polysaccharides for colon targeted drug delivery. Drug Deliv.

[3] Chaurasia M., Chourasia M.K., Jain N.K., Jain A., Soni V., Gupta Y., Jain S.K. (2006). Cross-linked guar gum microspheres: A viable approach for improved delivery of anticancer drugs for the treatment of colorectal cancer. AAPS PharmSciTech.

[4] Murthy S.N., Hiremath S.R.R., Paranjothy K.L.K. (2004). Evaluation of carboxymethyl guar films for the formulation of transdermal therapeutic systems. Int. J. Pharm.

[5] Cunha P.L.R., Castro R.R., Rocha F.A.C., de Paula R.C.M., Feitosa J.P.A. (2005). Low viscosity hydrogel of guar gum: Preparation and physicochemical characterization. Int. J. Biol. Macromol.

[6] Goycoolea F.M., Calderón de la Barca A.M., Balderrama J.R., Valenzuela J.R. (1997). Immunological and functional properties of exudate gum from northwestern Mexican mesquite (*Prosopis* spp.) in comparison with gum Arabic. Int. J. Biol. Macromol.

[7] Andrade C.T., Azero E.G., Luciano L., Gonçalves M.P. (1999). Solution properties of the galactomannans extracted from the seeds of *Caesalpinia pulcherrima* and *Cassia javanica*: Comparison with locust bean gum. Int. J. Biol. Macromol.

[8] Singh V., Srivastava A., Tiwari A. (2009). Structural elucidation, modification and characterization of seed gum from *Cassia javahiki* seeds: A non-traditional source of industrial gums. Int. J. Biol. Macro.

[9] Warrand J., Michaud P., Picton L., Muller G., Courtois B., Ralainirina R., Courtois J. (2005). Structural investigations of the neutral polysaccharide of *Linum usitatissimum* L. seeds mucilage. Int. J. Biol. Macromol.

[10] Estévez A.M., Saenz C., Hurtado M.L., Escobar B., Espinoza S., Suarez C. (2004). Extraction methods and some physical properties of mesquite (*Prosopis chilensis* (Mol) Stuntz) seed gum. J. Sci. Food Agric.

[11] Singthong J., Ningsanond S., Cui S.W. (2009). Extraction and physicochemical characterisation of polysaccharide gum from Yanang (*Tiliacora triandra*) leaves. Food Chem.

[12] Rana V., Rai P., Tiwary A.K., Singh R.S., Kennedy J.F., Knill C.J. (2011). Modified gums: Approaches and applications in drug delivery. Carbohydr. Polym.

[13] Ibaňez M.C., Ferrero C. (2003). Extraction and characterization of the hydrocolloid from *Prosopis flexuosa* DC seeds. Food Res. Int.

[14] Singh V., Singh S.K., Maurya S. (2010). Microwave induced poly (acrylic acid) modification of *Cassia javanica* seed gum for efficient Hg (II) removal from solution. Chem. Eng. J.

[15] Koocheki A., Kadkhodaee R., Mortazavi S.A., Shahidi F., Taherian A.R. (2009). Influence of *Alyssum homolocarpum* seed gum on the stability and flow properties of O/W emulsion prepared by high intensity ultrasound. Food Hydrocoll.

[16] Wu Y., Cui S.W., Tang J., Gu X. (2007). Optimization of extraction process of crude polysaccharides from boat-fruited *Sterculia* seeds by response surface methodology. Food Chem.

[17] Avallone R., Plessi M., Baraldi M., Monzani A. (1997). Determination of chemical composition of carob (*Ceratonia siliqua*): Protein, fat, carbohydrates, and tannins. J. Food Compos. Anal.

[18] Ho C.H.L., Cacacea J.E., Mazza G. (2007). Extraction of lignans, proteins and carbohydrates from flaxseed meal with pressurized low polarity water. LWT Food Sci. Technol.

[19] Piñeiro Z., Palma M., Barroso C.G. (2004). Determination of catechins by means of extraction with pressurized liquids. J. Chrom.

[20] Tabatabaee Amid B., Mirhosseini H., Kostadinovik S. (2012). Chemical composition and molecular structure of polysaccharide-protein biopolymer from *Durio zibethinus* seed: Extraction and purification process. Chem. Cent. J.

[21] Balaghi S., Mohammadifar M.A., Zargaraan A. (2010). Physicochemical and rheological characterization of gum tragacanth exudates from six species of Iranian *Astragalus*. Food Biophys.

[22] Razavi S.M.A., Mortazavi S.A., Matia-Merino L., Hosseini-Parvar S.H., Motamedzadegan A., Khanipour E. (2009). Optimisation study of gum extraction from Basil seeds (*Ocimum basilicum* L.). Int. J. Food Sci. Technol.

[23] Marcotte M., Taherian Hoshahili A.R., Ramaswamy H.S. (2001). Rheological properties of selected hydrocolloids as a function of concentration and temperature. Food Res. Int.

[24] McClements J. (2005). Food Emulsions: Principles, Practice, and Techniques.

[25] Peamprasart T., Chiewchan N. (2006). Effect of fat content and preheat treatment on the apparent viscosity of coconut milk after homogenization. J. Food Eng.

[26] Karazhiyan H., Razavi S.M.A., Phillips G.O. (2011). Extraction optimization of a hydrocolloid extract from cress seed (*Lepidium sativum*) using response surface methodology. Food Hydrocoll.

[27] Koocheki A., Mortazavi S.A., Shahidi F., Razavi S.M.A., Taherian A.R. (2009). Rheological properties of mucilage extracted from *Alyssum homalocarpum* seed as a new source of thickening agent. J. Food Eng.

[28] Ozkanli O. (2007). Evaluation of the Quality Parameters of Sumac Berries and Sumacs Concentrate. Ph.D. Thesis.

[29] Tuckova L., Novotna J., Novak P., Fleelova Z., Kveton T., Jelinkova L., Zidek Z., Man P., Tlaskalova-Hogenova H. (2002). Activation of macrophages by gliadin fragments: Isolation and charactderization of active peptide. J. Leukoc. Biol.

[30] Abd El-Hady E.A., Habiba R.A. (2003). Effect of soaking and extrusion conditions on antinutrients and protein digestibility of legume seeds. LWT Food Sci. Tecnol.

[31] Lopez da Silva J.A., Goncalves M.P. (1990). Studies on a purification method for locust bean gum by precipitation with isopropanol. Food Hydrocoll.

[32] Phimolsiripol Y., Siripatrawan U., Cleland D. (2011). Weight loss of frozen bread dough under isothermal and fluctuating temperature storage conditions. J. Food Eng.

[33] Singthong J., Ningsanond S., Cui S.W., Goff H.D. (2005). Extraction and physicochemical characterization of Krueo Ma Noy pectin. Food Hydrocoll.

[34] Glicksman M., Glicksman M. (1982). Functional properties of hydrocolloids. Food Hydrocolloids.

[35] Adewusi S.R.A., Osuntogun B.A. (1991). Effects of cooking on tannin content; trypsin inhibitor activity and *in vitro* digestibility of some legume seeds in Nigeria. Niger. Food J.

[36] Tabatabaee Amid B., Mirhosseini H. (2012). Optimization of aqueous extraction of gum from Durian (*Durio zibethinus*) seed: A potential, low cost source of hydrocolloid. Food Chem.

[37] Koocheki A., Taherian A.R., Bostan A. (2011). Studies on the steady shear flow behavior and functional properties of *Lepidium perfoliatum* seed gum. Food Res. Int..

[38] Pollard M.A., Kelly R., Wahl C., Windhab E., Eder B., Amado R. (2007). Investigation of equilibrium solubility of a carob galactomannan. Food Hydrocoll.

[39] Westphal O., Jann K. (1965). Bacterial lipopolysaccharides—extraction with phenol–water and further applications of the procedure. Method Carbohydr. Chem.

[40] Klahorst S. (2002). Beverage viscosity, Any way you like it. Food Prod. Des..

[41] Deis R. (2001). Salad dressings and sauces: Through thick and thin. Food Prod. Des..

[42] Tolstoguzov V.B. (1991). Functional properties of food proteins and role of protein—Polysaccharides interaction. Food Hydrocoll.

[43] Turgeon S.L., Beaulieu M., Schmitt C., Sanchez C. (2003). Protein—Polysaccharide interactions: Phase-ordering kinetics, thermodynamic and structural aspects. Curr. Opin. Colloid. Interface Sci.

[44] Nayebzadeh K., Chen J., Mousavi S.M.M. (2007). Interactions of WPI and xanthan in microstructure and rheological properties of gels and emulsions. Int. J. Food Eng.

[45] Zárate-Ramírez L.S., Bengoechea C., Cordobés F., Guerrero A. (2010). Linear viscoelasticity of carob protein isolate/locust bean gum blends. J. Food Eng.

[46] Abassi S., Dickinson E. (2004). Gelation of iota-carrageenan and micellar casein mixtures under high hydrostatic pressure. J. Agric. Food Chem.

[47] Bressani R., Benavides V., Acevedo E., Oritiz M.A. (1990). Changes in selected nutrient content and in protein quality of common and quality protein maize during torilla preparation. Cereal Chem.

[48] Laaman T.R. (2011). Hydrocolloids: Fifteen practical tips. Hydrocolloids in Food Processing.

[49] Tabilo-Munizaga G., Barbosa-Canovas G.V. (2005). Rheology for the food industry. J. Food Eng.

[50] Simas-Tosin F.F., Barraza R.R., Petkowicz C.L.O., Silveira J.L.M., Sassaki G.L., Santos E.M.R., Gorin P.A.J., Iacomini M. (2010). Rheological and structural characteristics of peach tree gum exudates. Food Hydrocoll.

[51] Mandala I.G., Savvas T.P., Kostaropoulos A.E. (2004). Xanthan and locust bean gum influence on the rheology and structure of a white model-sauce. J. Food Eng.

[52] Wang Y., Wang L., Li D., Ozkan N., Chen X.D., Mao Z. (2008). Effect of flaxseed gum addition on rheological properties of native maize starch. J. Food Eng.

[53] Tabatabaee Amid B., Mirhosseini H. (2012). Effect of different purification techniques on characteristics of heteropolysaccharide-protein biopolymer from durian *(Durio zibethinus*) seed. Molecules.

[54] Nakauma M., Funami T., Noda S., Ishihara S., Al-Assaf S., Nishinari K., Phillips G.O. (2008). Comparison of sugar beet pectin, soybean soluble polysaccharide, and gum arabic as food emulsifiers. 1. Effect of concentration, pH, and salts on the emulsifying properties. Food Hydrocoll.

[55] Tabatabaee Amid B., Mirhosseini H. (2012). Influence of different purification and drying methods on rheological properties and viscoelastic behaviour of durian seed gum. Carbohydr. Polym.

[56] Oomah D.B., Mazza G. (2001). Optimization of a spray drying process for flaxseed gum. Int. J. Food Sci. Technol.

